# Temporal adaptation of sensory attenuation for self-touch

**DOI:** 10.1007/s00221-023-06688-5

**Published:** 2023-08-22

**Authors:** Clara Fritz, Eckart Zimmermann

**Affiliations:** Institute for Experimental Psychology, Heinrich Heine University Düsseldorf, Düsseldorf, Germany

**Keywords:** Sensory attenuation, Efference copy, Self-touch, Temporal adaptation

## Abstract

The sensory consequences of our actions appear attenuated to us. This effect has been reported for external sensations that are evoked by auditory or visual events and for body-related sensations which are produced by self-touch. In the present study, we investigated the effects of prolonged exposure to a delay between an action and the generated sensation on sensory attenuation for self-touch. Previously, it has been shown that after being presented to a systematic exposure delay, artificially delayed touch can feel more intense and non-delayed touches can appear less intense. Here, we investigated the temporal spread of the temporal recalibration effect. Specifically, we wondered whether this temporal recalibration effect would affect only the delay that was used during exposure trials or if it would also modulate longer test delays. In the first two experiments, we tested three test delays (0, 100 and 400 ms) either in randomized or in blocked order. We found sensory attenuation in all three test intervals but no effect of the exposure delay. In Experiment 3, we replicated the experiment by Kilteni et al. (ELife 8:e42888, 2019. 10.7554/eLife.42888) and found evidence for temporal recalibration by exposure delay. Our data show that the temporal selectivity of sensory attenuation of self-touch depends on presenting a singular test delay only. Presenting multiple test delays leads to a temporally broad spread of sensory attenuation.

## Introduction

Imagine moving your right finger to touch your left arm. Even before your finger makes contact, your brain knows what you are going to sense. Sensorimotor predictions lead to a decrease in the perceived intensity of touch, a phenomenon termed sensory attenuation (Blakemore et al. 1998, 1999). The most prominent example for sensory attenuation is the inability to tickle ourselves. Self-produced touches are perceived as weaker or less intense than externally produced touches (von Holst and Mittelstaedt 1950). The standard theory of sensory attenuation is the comparator model that relies on the movement generation architecture provided by classical control theory (Blakemore et al. 2000, 2002). In this scheme, a movement plan is constructed by an inverse model. The movement plan is sent to the motor plant in order to relay a movement command to the muscles. A copy of the movement plan, the so-called efference copy is used to compute a forward model that entails a prediction of the sensory consequences following the planned movement. When the movement is finished, the predicted and the actual sensory consequences are compared. If the prediction matches the actual consequences, the sensation is attenuated (Bays et al. 2005, 2006, 2008; Witney et al. 1999; Wolpert and Flanagan 2001).

Kilteni et al. (2020) demonstrated that sensory attenuation only occurs when performing an active, compared to a passive self-generated touch. In their experiment, participants were asked to actively press a force sensor with their right index finger after hearing an auditory go signal. The press resulted in a lever touching the top of their left index finger. In a second condition, participants placed their right index finger on a plastic surface, located above the force sensor for the right index finger. When the auditory go signal occurred the plastic surface vanished, causing participants finger to fall freely onto the underlying sensor. Again, this tap resulted in a lever touching the top of their left index finger. Although the contact by the lever was self-generated and predictable in both conditions, sensory attenuation was only evident in the active condition. In addition, the extent of the perceived intensity of the lever pressure in the passive condition was comparable to that of an externally generated contact. The authors concluded that an active, self-generated movement produces an efference copy that is responsible for the attenuation effect of self-contact. In accordance with the comparator model the findings suggest that a small prediction error (coupled with a higher accuracy of prediction) will lead to a less intensely perceived touch compared to a touch resulting from an external movement (Blakemore et al. 1998; Fraser and Fiehler 2018; Voudouris and Fiehler 2017, 2021). Importantly, touch is spatially selective for the goal location of the touching movement (Bays et al. 2008; Kilteni and Ehrsson 2017; Knoetsch and Zimmermann 2021). Such a precision requires that the mechanism, inducing sensory attenuation, takes into account a detailed description of the positions of body parts.

If systematic changes in the timing between a motor action and the corresponding sensory feedback occur, the sensorimotor system adapts (Cunningham et al. 2001; Heron et al. 2009; Rasman et al. 2021). This recalibration process has been studied by asking subjects to judge the temporal order between a button press and a flash (Stetson et al. 2006). When the authors injected a fixed delay, temporal order judgements shifted toward the injected delay. A following study determined that it is the motor component which temporally shifts toward the perceptual event (Sugano et al. 2010). Adaptation to temporal delays between action and perception has also been investigated in the multi-sensory domain. Data have suggested that auditory feedback for motor-sensory temporal recalibration is more likely to occur than for visual feedback (Sugano et al. 2016). Furthermore, recalibration transferred from vision to audition but not vice versa (Arikan et al. 2021). Other studies found that performing actions widens the window of multi-sensory simultaneity, irrespective of whether the movement is voluntary or involuntary (Arikan et al. 2017). The window of audio-visual simultaneity can be increased when participants learn that the delay between action and audio-visual pair is variable (Desantis and Haggard 2016). Results from our lab have recently shown that the strength of sensory attenuation for visual events is modulated by injected delays between action and perception (Storch and Zimmermann 2022).

Kilteni et al. (2019) asked whether temporal recalibration could also be demonstrated for sensory attenuation for self-touch. In their experiment participants had to touch a force sensor with their right finger in order to rotate a lever to tap their left finger. A systematic delay of 0 ms or 100 ms (exposure delay) was inserted between the voluntary tap of the right index finger and the resulting touch on the pulp of the relaxed left index finger. After exposing subjects for 500 trials to a 0 ms delay between button press and lever touch, they found sensory attenuation in test trials when the lever touch followed immediately after button press (0 ms test delay), but not when it followed 100 ms later (100 ms test delay). The crucial condition contained an exposure of 500 trials to a delay of 100 ms between button press and lever touch. In these sessions, subjects were supposed to learn a new temporal sensorimotor contingency between button press and the time of the ensuing tactile sensation. After being exposed to the 100 ms exposure delay, subjects showed no sensory attenuation in test trials, which contained 0 ms between button press and tactile sensation (test delay 0 ms). This absence of sensory attenuation was interpreted by the authors (Kilteni et al. 2019) as unlearning of sensory attenuation. In contrast, subjects were able to learn attenuated touch based on the predicted delay of 100 ms.

Here, we sought to investigate first whether temporal recalibration leads to attenuation of touches presented at the same test delay as the exposure one or if attenuation will be observed for touches presented at other delays too. In the latter case, prolonged exposure to a delay in self-touch might also be observable in test delays which are longer than the exposure delay. Second, we asked if the predictability of an experimental condition e.g., a learned test delay of 0 ms or 100 ms, might influence the ability of learning a prolonged delay. We asked whether blocked versus randomized presentation of test delays show different effects. To investigate this hypothesis, we tested our introduced test delays of a non-movement baseline, 0 ms, 100 ms and 400 ms in a randomized and separately in a blocked order. We expected that the effects of temporal predictability would no longer occur, when comparing sensory attenuation magnitudes for different probe delays. We also conducted a replication of one of the experiments by Kilteni et al. (2019).

## Methods

### Participants

Participants were recruited in the University Düsseldorf, by personal contacts or via social networks. All experiments were in accordance with the Declaration of Helsinki and were approved by the local ethics committee of the Faculty of Mathematics and Natural Sciences of Heinrich Heine University, Düsseldorf (Identification No. 757184). Handedness was assessed using the Edinburgh Handedness Inventory and written informed consent was obtained. Participants were compensated with participation hours or remunerated by means of an expense allowance.

### Experimental setup

Participants were seated in front of a table in a quiet room with the apparatus placed in front of them. Participants placed their left hand in an upside-down position approximately 5 cm underneath a motor (Savöx SC-1257 TG). The motor was mounted underneath a metal arc with the help of double-sided adhesive tape. The left index finger was touched by the motor only when moving the lever. To keep the left forearm and hand comfortable bubble wrap was used. The right hand laid on top of the metal arch with the right hand resting above of a force sensor (FSR^®^, Interlink Electronics, Inc, Camarillo, CA 93012, USA). Participants were instructed to press the force sensor with their right index finger only.

**Fig. 1:**
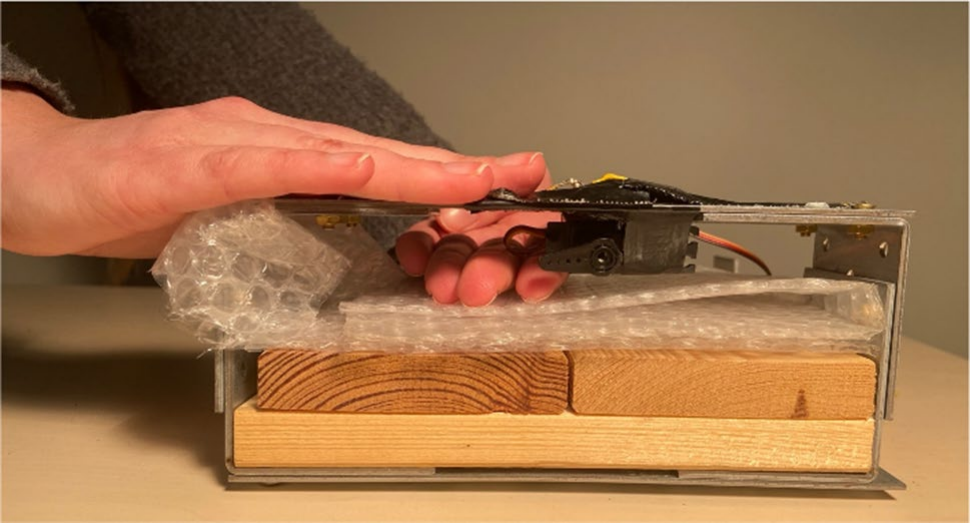
Experimental setup. The left hand was placed underneath the motor which was attached to a metal arch. Once the auditory go signal occurred, participants were instructed to press the force sensor with the right index finger which led to the left index finger being touched by a lever controlled by the motor

In exposure trials, the apple system sound “Funk” was used as the auditory go signal (duration: 510 ms, frequency: 44.1 kHz). In test trials, a tom tom drum sound was used (duration: 250 ms, frequency: 44.1 kHz). After pressing the force sensor, the motor’s lever simulated a finger touch to the left index finger. The motor was controlled by a custom-made Objective-C program, by sending commands via the serial port to the micro-controller (Arduino Nano Atmega328 CH340). The Arduino Nano was connected to a MacBook Pro (Retina, 15-inch, 2015). Since information within the micro-controller is processed in the micro-seconds range, the delay between force sensor presses and motor rotation is mostly produced by the regulating time of the motor. The Savöx SC-1257 TG when used at 4.8 V (in our study 5 V was used) has a regulating time of 90 ms/60°. We let the motor rotate the connected lever by 20°. There was thus an approximate delay between button press and lever rotation of 30 ms. The duration of a tactile stimulation was 270 ms in Experiment 1 and 2 and 100 ms in Experiment 3. The experimental setup can be seen in Fig. 1.

During the experiments, we differentiated between exposure trials and test trials. In exposure trials, subjects heard an auditory go signal which indicated to press the force sensor. After pressing, the lever rotated with a delay of either 0 ms or 100 ms to simulate a touch on participant’s left index finger. In test trials, subjects heard a different auditory go signal (tom tom drum) to sensitize them for a response. During test trials, the lever rotated two times after the force sensory was pressed. The first rotation (probe touch) occurred with either − 100 ms (baseline), 0 ms, 100 ms or 400 ms delay (depending on the experiment). The second rotation (reference touch) occurred automatically 1500 ms after the probe touch. Participants were asked to indicate whether the first or the second touch felt stronger. Therefore, we used two-alternative forced choice (2AFC), so participants were forced to decide between the stimuli. Responses were made by the help of a foot pedal (UPC ECS-PPD-F) placed under the table.

### Experiment 1

In Experiment 1, we tested 36 participants (15 male, 21 female). Two of them were left-handed and their age ranged from 18 to 37 years (*M* = 25.63).

The experiment started with 500 exposure trials. 1000 ms after trial start an auditory cue (Apple System Sound) indicated to press the force sensor. Once the micro-controller detected that the force sensor was pressed, the lever was rotated by the motor and touched the subjects right index finger with a strength of subjectively perceived 2 N. Depending on the exposure delay, the rotation occurred either simultaneously with the press or 100 ms later. Exposure delays were tested in different sessions. Thus, in Experiment 1, participants had to perform two sessions: either with an introduced exposure delay of 0 ms or 100 ms. We randomized the order of sessions.

In test trials, the auditory go signal consisted of a tom tom drum sound. When pressing the force sensor during a test trial, the lever rotated two times. The rotation of the first, the probe touch, occurred either simultaneously with the press or after a certain delay (test delay). The test delay varied between conditions, with the lever rotating either 100 ms before the auditory signal (baseline), directly with the force sensory press (0 ms), 100 ms or 400 ms after the force sensory press. In the baseline condition, participants did not have to press the force sensor to trigger a movement of the motor. 100 ms before the auditory go signal occurred, the lever rotated automatically. The four conditions (baseline, test delay 0, 100 and 400 ms) were presented in randomized order for each of the two exposure delays. The 0 ms and the 100 ms exposure delays were measured in separate sessions.

**Fig. 2:**
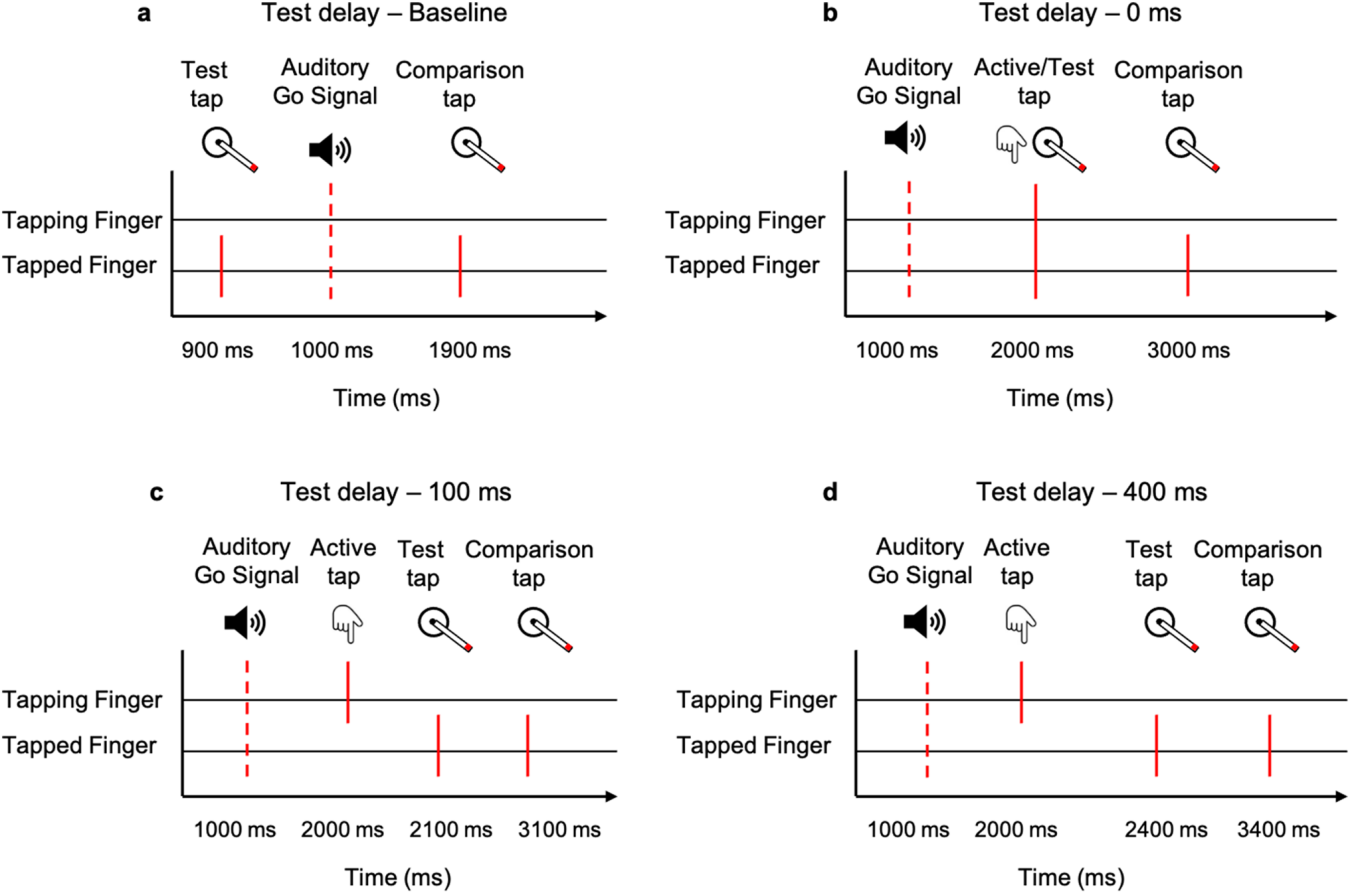
Temporal outline of test delays in Experiments 1 and 2. Participants heard an auditory go signal via headphones, indicating them to press the force sensor. During test trials, the delay between active tap and test tap varied between 0, 100, 400 ms or no active tap had to be conducted to receive a test tap baseline). The comparison tap occurred 1500 ms after the test tap.

After the probe touch, a second, the reference touch occurred 1500 ms later on the same finger. As stated above participants were forced to decide which touch felt stronger (2AFC). When participants gave their response, the next trial started immediately. Each test trial was followed by 5 exposure trials, then the next test trial was presented again. In Experiment 1, we had 80 test trials in total. As one experimental session started with 500 exposure trials, followed by 1 test trial and 5 exposure trials again, we had a total of 980 trials in each session. As stated above, for Experiment 1, two sessions were performed, so we had a total of 1960 trials. The temporal outline of the exposure and test delays can be seen in Fig. 2.

An adaptive staircase procedure (Best PEST) was used to estimate the perceived equality of probe and reference touch (Pentland 1980). The Best PEST method is an adaptive method of psychophysics for determining the perception threshold of a subject to a stimulus. In our experiment, threshold estimation was conducted between subjectively perceived 1.7–2.3 N in steps of 0.1 N. Subjectively perceived N values for the applied motor force were determined with the help of a short pilot testing in advance to testing experiments. We asked a different data set of participants to press the force sensor with their right index finger. The intensity of the press was read out by the connected micro-controller and displayed on the computer. We could thus assess the exact force applied during pressing. For each a priori determined increment between 1.7 and 2.3 N, subjects were asked to press the sensor with the corresponding strength. After an individual training period, subjects were able to follow instructions. For each press the motor lever provided a certain force on the left index finger of the subjects. They could adjust the force of the motor gradually until they were confident that it matched the force of their press. Progression of the pressed strength and the output received is linear. With this procedure, we could produce an average mapping between the a priori defined increments (1.7–2.3 N) and the subjectively determined forces that the motor has to apply. After pilot testing, we averaged subjectively perceived values that were used as standard values across participants for the following experiments. The values remained identical except for differences between Experiment 1 and 2 versus 3. The presentation of the next stimulus increments depended on the subject's previous response. Participants used the foot pedal to indicate which of two stimuli felt stronger. Based on this response, the subsequent stimulus was adjusted. If the previous response resulted in a stronger perception of the stimulus, the subsequent stimulus strength was adjusted correspondingly lower. Conversely, if the previous response reported a weaker perception of the stimulus, the subsequent stimulus strength was increased. Thus, the stimulus strength adapts to a threshold over time.

As the Best PEST method offers an adaptive parameterization of stimuli, this method increases accuracy and the elimination of systematic errors. Less trials for the analysis are needed, reducing the number of stimulus presentations and thus the duration of the experiment (Lieberman and Pentland 1982).

### Experiment 2

In Experiment 2, we collected data of 40 participants. Two subjects had to be excluded due to wrong task execution. Therefore, in the analysis, we included 38 subjects (16 male, 21 female, 1 diverse) with a mean age of *M* = 24.79 and age ranging from 18 to 36 years. Three subjects were left-handed.

The methodological structure of Experiment 2 was similar to Experiment 1. Again, subjects were asked to conduct two sessions, with a presented exposure delay of either 0 or 100 ms in the exposure trials. It was randomized which exposure delay occurred first. Though, here the four test delays (baseline, 0 ms, 100 ms, 400 ms) were presented block-wise. In Experiment 1, the test delays were presented randomly, whereas in this experiment, the same test delay was presented for 20 test trials. Otherwise, the trial structure remained similar to Experiment 1: at the beginning, 500 exposure trials were presented. Afterwards, one test trial was followed by 5 exposure trials again. For every test delay, 20 test trials were presented, so we had 80 test trials in total, having 980 trials in total per session. As participants had to conduct one session with an exposure delay of 0 ms and one with an exposure delay of 100 ms, 1960 trials were conducted per participant.

### Experiment 3

Experiment 3 included data of 36 participants. We tested 9 male and 27 female with a mean age of *M* = 24.66 and age racing from 18 to 37 years. The task as well as the exposure delays in Experiment 3 were identical to Experiment 1 and 2. Sessions were conducted with an exposure delay of either 0 ms or 100 ms. For the test delays, we included only 0 ms and 100 ms. Experiment 3 contained 4 conditions: an exposure delay of 0 ms and a test delay of 0 ms/100 ms or an exposure delay of 100 ms and a test delay of 0 ms/100 ms. We measured every condition in a separate session, so participants had to complete 4 sessions in total in Experiment 3. The order or conditions was randomized between test subjects. In Experiment 3, a psychometric function with 7 constant stimuli was measured. The probe touch had a strength of subjectively perceived 2 N. To this end, the strength of the second reference touch by the lever in a given trial was chosen randomly out of 7 possible reference magnitudes (subjectively perceived 1.4–2.6 N in 7 equidistant steps). For every reference magnitude, 10 test trials were presented. Tactile stimulation lasted 100 ms. Every test trial was followed by 5 exposure trials. The experiment started with 505 exposure trials and participants had to complete 70 test trials and 350 extra exposure trials.

### Power analysis

A statistical power analysis was performed for sample size estimation, based on data from Kilteni et al. (2019) (*N* = 30). The effect size in the study of Kilteni et al. (2019) varied between conditions (ranging from 0.343 to 0.848). As we were looking predominantly on the classical effect of expected versus unexpected delays, we conducted a power analysis for the effect sizes of the paired *t*-test between (exposure delay: 100 ms, test delay: 100 ms) and (exposure delay:  0 ms, test delay: 100 ms): *t*(29) = − 3.29, *p* = 0.003, *CI*^*95*^ = [− 0.142,–0.033], *d* = 0.601. With an *α* = 0.05 and power = 0.95, the projected sample size needed with this effect size is approximately *N* = 38. Thus, our proposed sample size should be adequate for the main objective of this study and should also allow for expected attrition.

## Results

### Data analysis

Since the Best PEST method uses an adaptive threshold procedure, participant’s individual perceived intensity of touch [N] is revealed towards the end of the test trials. We used 20 test trials for each threshold determination and used the last 5 values to calculate thresholds. In Experiments 1 and 2, four thresholds were measured per session per condition (for baseline, 0 ms, 100 ms, 400 ms). Since we conducted two sessions in each experiment to test the different exposure delays (0 ms, 100 ms), this resulted in eight thresholds per subject in the respective experiment.

For Experiment 3, data were fitted by a cumulative Gaussian function for each condition and test delay per participant (cumulative Gaussian function, fcn = @(*b*, *x*) normcdf(*x*, *b*(1), *b*(2)); NRCF = @(*b*) norm(Y/100—fcn(*b*, *X*)); B = fminsearch(NRCF, [0; 10], fitted in MATLAB_R2020b). The point of subjective equality (PSE) represents the magnitude at which the probe tap is perceived as stronger than the comparison tap on 50% of the trials.

Data were analyzed in JASP 0.16.3 and MATLAB_R2020b.

### Experiment 1

For Experiment 1, we estimated the subjective intensity [N] of a self-touch with an adaptive staircase method (Best PEST). Estimated intensity values were then averaged for each condition across all subjects. A repeated-measures 2 × 4 ANOVA was conducted to identify whether the conditions were significantly different from each other. The ANOVA included the two factors exposure delay (with levels 0 ms and 100 ms) and test delay (with levels of four test delays [Baseline, 0 ms, 100 ms, 400 ms]).

**Fig. 3:**
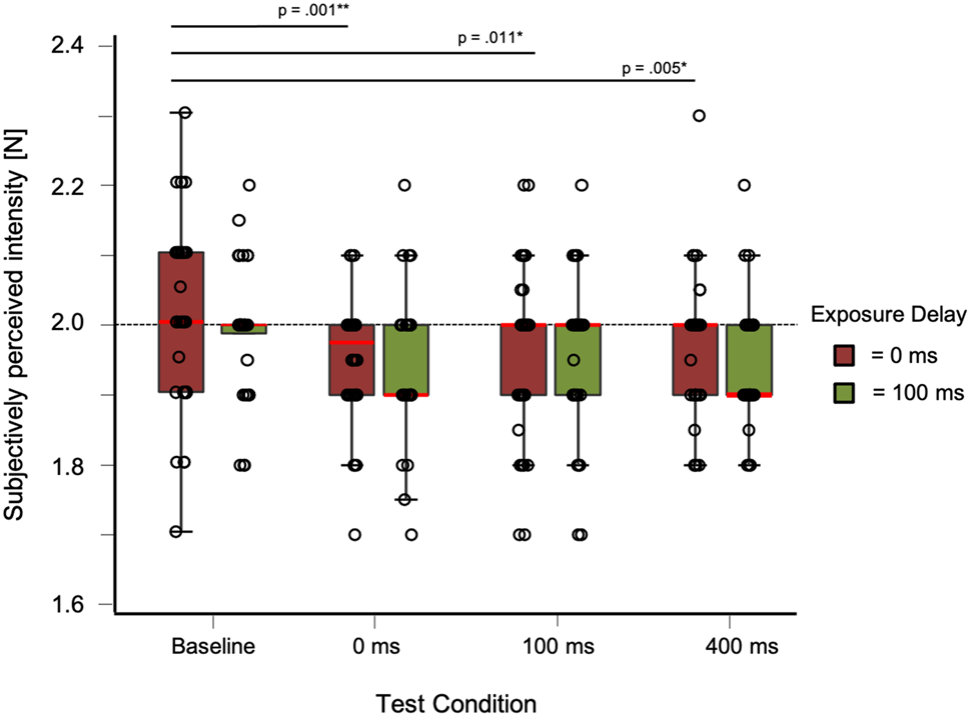
Box-and-whisker plots for Experiment 1. Tests conditions are shown against subjectively perceived intensity [N] including individual data points. Red bars for each condition represent the median

Descriptive statistics for the test trials can be found in Table 1 and distribution of data in Fig. 3.

**Table 1:** Mean and standard deviation for test trials in Experiment 1

Exposure delay (ms)	Test delay							
	Baseline		0 ms		100 ms		400 ms	
	*M*	SD	*M*	SD	*M*	SD	*M*	SD
0 ms	2.02	0.13	1.95	0.09	1.96	0.12	1.96	0.10
100 ms	1.99	0.09	1.95	0.10	1.96	0.11	1.95	0.09

The repeated-measures ANOVA showed no significant effects for the factor exposure delay (*F*(1, 35) = 0.819, *p* = 0.372). As the Mauchly-Test was significant, the Greenhouse–Geisser adjustment was used to correct for violations of sphericity in the condition test delay and the interaction. All other requirements for parametric testing were met. The interaction showed no significant effect (*F*(2.65, 35) = 0.39, *p* = 0.76). For the factor test delay, we found a significant effect (*F*(2.36, 35) = 6.27, *p* = 0.002).

**Fig. 4:**
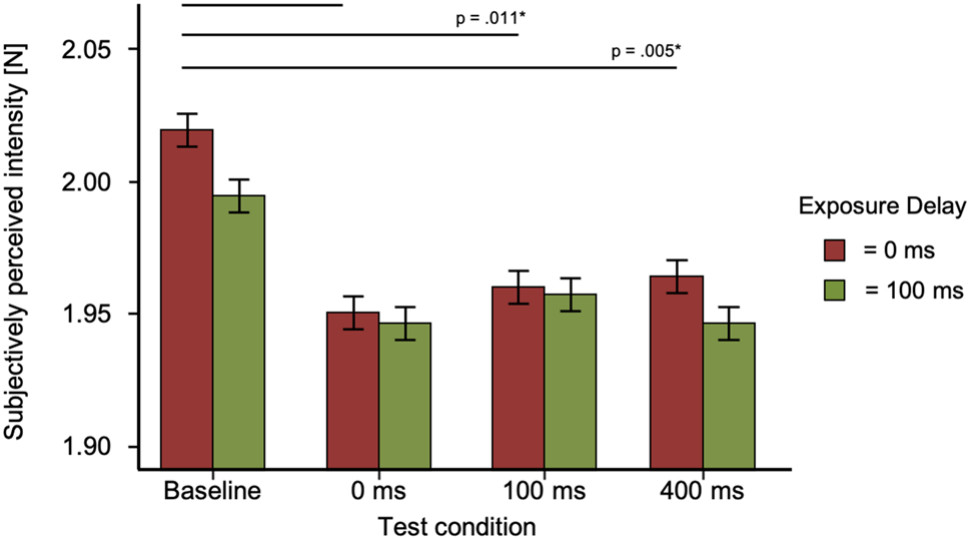
Results of Experiment 1. Bar plots for every exposure and test trial. Tests conditions are shown against subjectively perceived intensity [N]. Error bars represent standard errors (s.e.m.)

A post hoc analysis for the factor test delay (tested against a Bonferroni-adjusted alpha level of 0.05/6) revealed that there was a significant difference between the test delay of baseline and 0 ms (MD = 0.06, SE = 0.02, *p* = 0.001, *d* = 0.646) as well as a test delay of baseline and 100 ms (MD = 4.86, SE = 1.523, *p* = 0.011, *d* = 0.532) and baseline and 400 ms (MD = 5.21, SE = 1.523, *p* = 0.005, *d* = 0.570). *p* values are Bonferroni corrected (*p*/6). No significant effects were found between the other delays (*p* > 0.81). Distribution of data between test conditions for Experiment 1 is shown in Fig. 4.

In Experiment 1, test conditions were randomized within each session so timing of the presented test delay was not predictable. In order to test whether the absence of effects of the exposure delay occurred due to the missing temporal predictability, we conducted a second experiment in which we presented the test intervals in four fixed blocks.

### Experiment 2

**Fig. 5:**
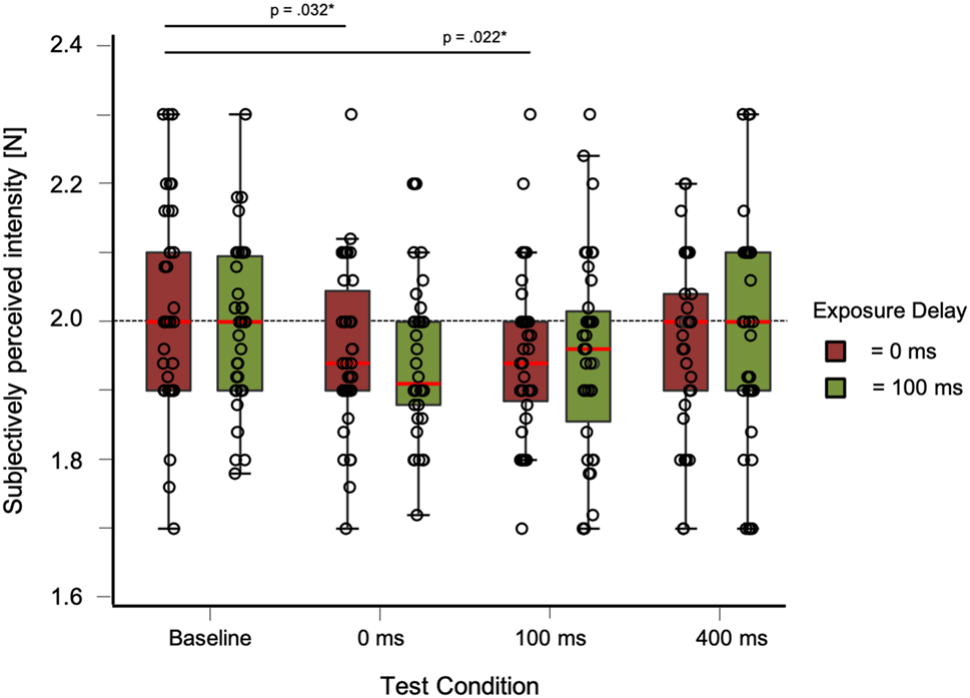
Box-and-whisker plots for Experiment 2. Tests conditions are shown against subjectively perceived intensity values [N] including individual data points. Red bars for each condition represent the median

In Experiment 2, we presented test trials in a blocked order. Each test delay was presented for 20 test trials in total, intermixed with 5 exposure trials after each test trial. The order of test delay blocks was counterbalanced across participants. As the overall method remained identical, we conducted the same steps for data analysis as in Experiment 1: the last 5 stimuli values for each test and exposure delay per participant were averaged. We also conducted a repeated-measures 2 × 4 ANOVA (factor one: exposure delay of 0 ms and 100 ms, factor two: test delays of baseline, 0 ms, 100 ms, 400 ms) and used paired sample *t*-tests for comparison between baseline and the other conditions. The mean and standard deviation for the different exposure and test delays are shown in Table 2. Figure 5 shows distribution of data for the conditions.

**Table 2:** Mean and standard deviation for test trials in Experiment 2

Exposure delay (ms)	Test delay							
	Baseline		0 ms		100 ms		400 ms	
	*M*	SD	*M*	SD	*M*	SD	*M*	SD
0 ms	2.02	0.15	1.96	0.12	1.95	0.12	1.97	0.13
100 ms	1.99	0.12	1.95	0.12	1.95	0.15	1.98	0.16

**Fig. 6:**
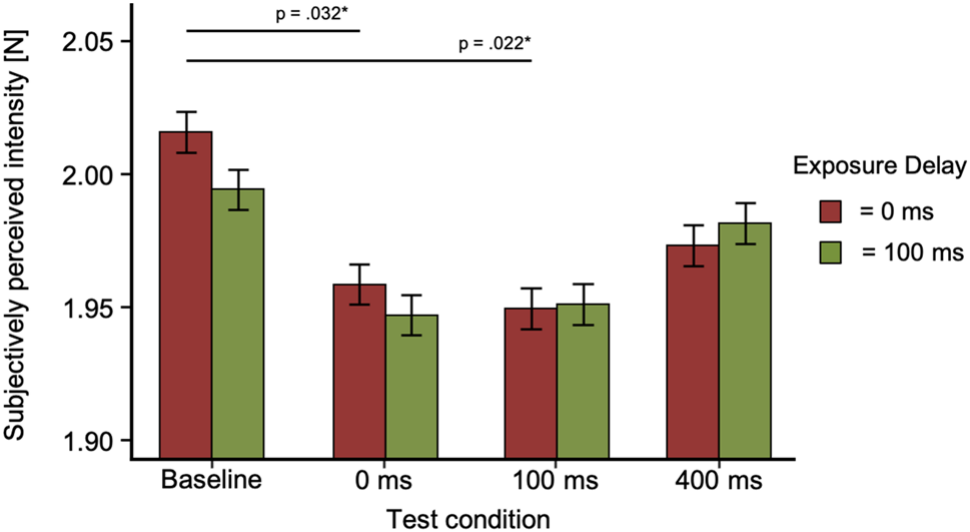
Results of Experiment 2. Bar plots for every exposure and test trial. Tests conditions are shown against subjectively perceived intensity [N]. Error bars represent standard errors (s.e.m.)

As the Mauchly-Test was significant, the Greenhouse–Geisser adjustment was used to correct for violations of sphericity in the condition test delay and the interaction. All other requirements for parametric testing were met. The ANOVA showed a significant main effect for the factor test delay (*F*(3, 37) = 3.86, *p* = 0.016, $n_{p}^{2}$ = 0.041). For exposure delay (*F*(3, 37) = 0.095, *p* = 0.76) and exposure delay *x* test delay (*F*(3, 37) = 0.359, *p* = 0.777) no significant effects were found. A post hoc analysis revealed that there was a significant difference between the test delay of baseline and 0 ms (MD = 0.05, SE = 0.18, *p* = 0.032, *d* = 0.396) as well as a test delay of baseline and 100 ms (MD = 0.06, SE = 0.18, *p* = 0.022, *d* = 0.414). *p* values are Bonferroni corrected (*p*/6). No significant effects were found between the other delays (*p* > 0.817). Figure 6 shows an overview of results.

We found weaker effects between conditions compared to Experiment 1. We aimed to rule out that differences to the results of the original study of Kilteni et al. (2019) were due to the choice of the threshold estimation method. Kilteni et al. (2019) used psychometric functions, whereas we used an adaptive staircase (Best PEST) procedure. To this end, we measured subjects with psychometric functions as well and conducted a conceptual replication of Kilteni et al. (2019).

### Experiment 3

**Fig. 7:**
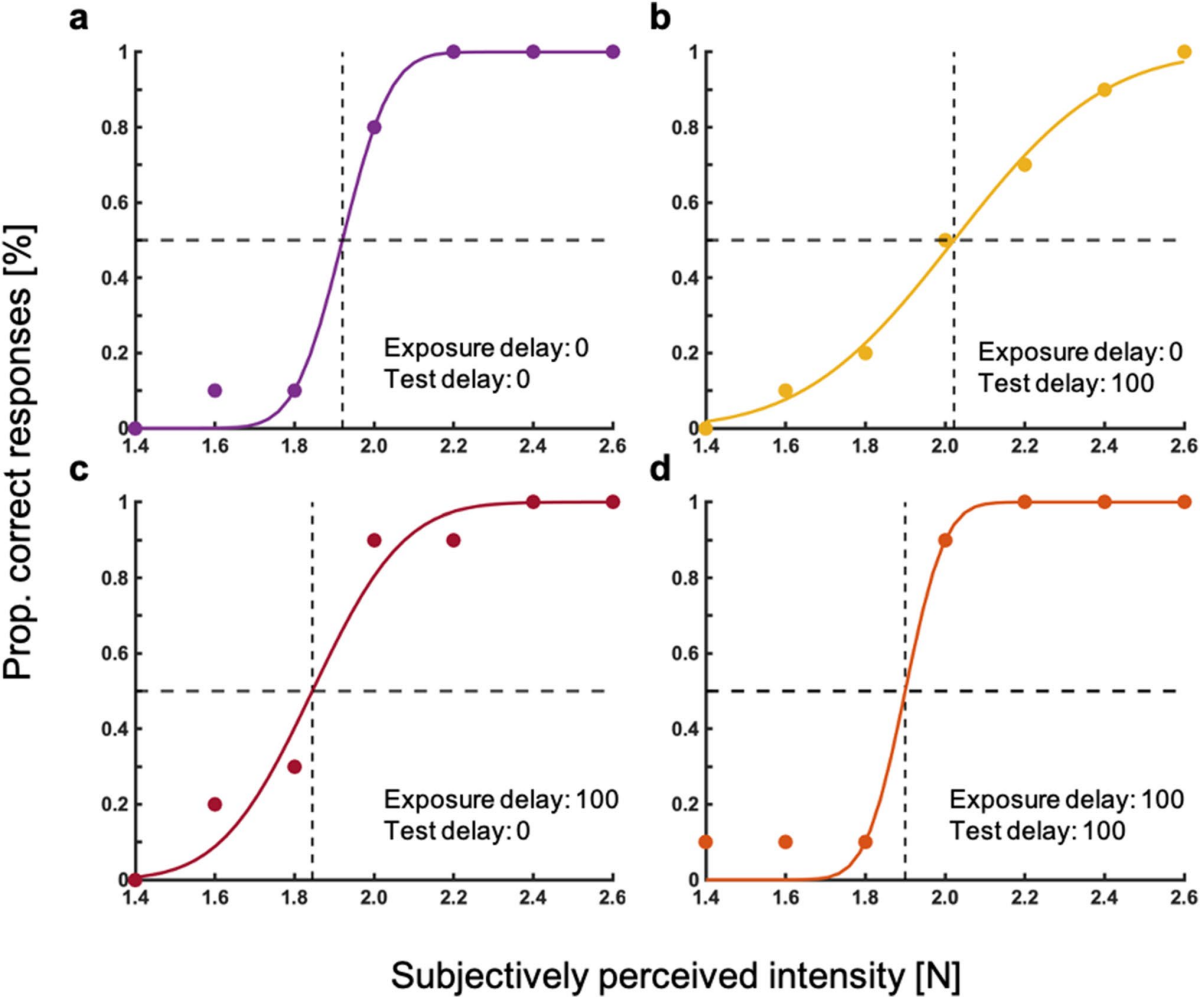
Psychometric functions of an example participant for the four conditions. Proportions of correct responses are shown against the perceived intensity of second touch

**Fig. 8:**
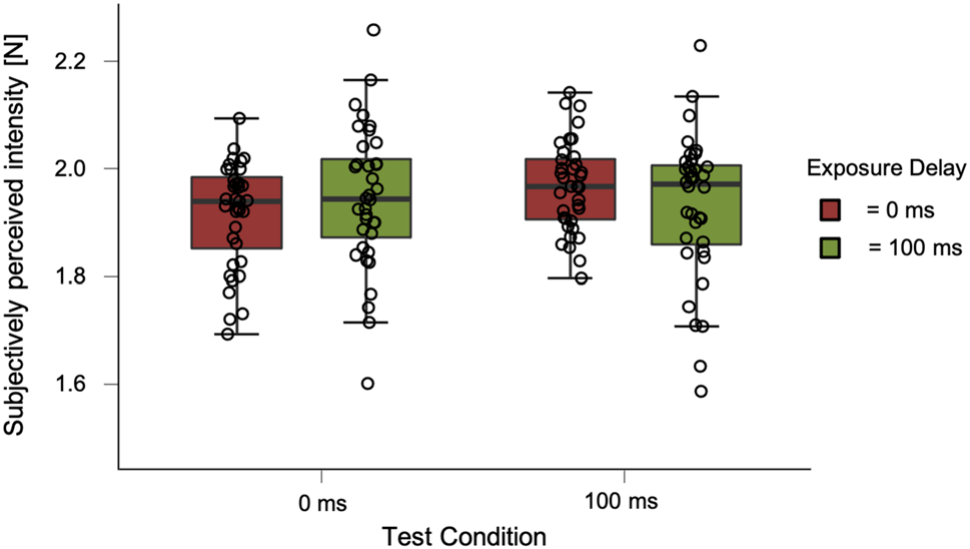
Box-and-whisker plots for Experiment 3. Tests conditions are shown against subjectively perceived intensity [N] including individual data points. Black bars for each condition represent the median

In Experiment 3, we tested the same conditions as in Kilteni et al. (2019). Data were analyzed with psychometric functions seen in Fig. 7. Similar to the other experiments, the exposure delay differed between no delay (0 ms) and 100 ms delay during exposure trials. The factor test delay differed between a 0 ms or 100 ms delay of the test touch after the force sensor press. Mean and standard deviation for PSE and JND of the experiment are shown in Table 3. Data distribution is shown in Fig. 8.

**Table 3:** Mean and standard deviation for PSE and JND in Experiment 3

Exposure delay (ms)	Test delay							
	0 ms (PSE)		100 ms (PSE)		0 ms (JND)		100 ms (JND)	
	*M*	SD	*M*	SD	*M*	SD	*M*	SD
0 ms	1.92	0.10	1.97	0.09	0.25	0.14	0.23	0.13
100 ms	1.95	0.13	1.93	0.13	0.23	0.12	0.26	0.13

**Fig. 9:**
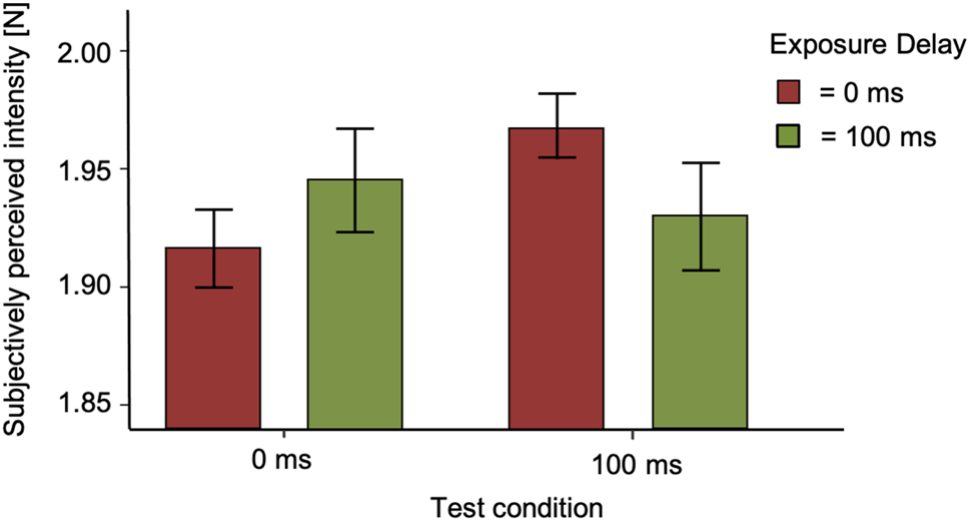
Results of Experiment 3. Bar plots for the two test conditions and exposure delays. Tests conditions are shown against subjectively perceived intensity [N]. Error bars represent the standard error of the mean

As the requirements for parametric testing were met, we conducted a 2 × 2 repeated-measures ANOVA (factor one: exposure delay of 0 and 100 ms, factor two: test delay of 0 ms and 100 ms). No significant effects for either the factor exposure delay (*F*(1, 35) = 0.063, *p* = 0.803) nor test delay (*F*(1, 35) = 1.783, *p* = 0.190) were found. The interaction between exposure delay and test delay was significant (*F*(1, 35) = 4.869, *p* = 0.034). Bar plots can be seen in Fig. 9.

Since the authors in the original study of Kilteni et al. (2019) used a priori planned paired *t*-tests to compare effects of sensory attenuation between conditions, we checked our data for the effect of classical sensory attenuation with this analysis method. We conducted a paired sample *t*-test between the test conditions of an exposure and test delay of 0 ms compared to an exposure delay of 0 ms and a 100 ms test delay (*t*(35) = − 2.86, *p* = 0.007, *d* = 0.476).

## Discussion

In this study, we asked about the spread of temporal recalibration in sensory attenuation of self-touch. When participants are asked to touch their left with their right finger and, by means of an experimental device, are exposed over several trials to a delay (100 ms) between their touch and the ensuing tactile sensation, sensory attenuation shifts in time to the exposed delay (Kilteni et al. 2019). Furthermore, sensory attenuation disappeared when self-touch was tested without a temporal delay. These data are consistent with a temporal recalibration mechanism which adapts the internal prediction of the time when actions yield sensory consequences.

We wondered whether temporal recalibration would tightly couple sensory attenuation to the newly learned delay (100 ms) or if it would spread more broadly when tested in longer intervals. The first possibility would be in line with a very precise temporal recalibration whereas the latter would argue for a shift of the entire tuning curve of sensory attenuation. Previous research investigating the time-course of sensory attenuation of self-touch found that the decrease in perceived tactile intensity starts 300 ms before the active right finger touches the passive left finger and returns to baseline level about 300 ms after it (Bays et al. 2005). We reasoned that adaptation to a delay between the active touch and the passive tactile sensation might shift this tuning curve rather broadly such that sensory attenuation could even be measured at longer delays. To this end, in our first two experiments, we tested three temporal delays (0, 100 and 400 ms) after separate exposure delays of 0 ms and 100 ms. In Experiment 1, these test intervals were randomly interleaved between exposure trials. The ratio of the number of exposure and test trials was identical to previous studies (Stetson et al. 2006; Kilteni et al. 2019). To our own surprise, we found sensory attenuation in all test delays and no effect of the exposure delays. In order to rule out that the randomly interleaved presentation of three different test delays might have led to this broad spread of sensory attenuation, we conducted a second experiment, in which we blocked the presentation of test delays. The data in this experiment were similar to the first experiment.

To better understand these results, in our Experiment 3, we replicated the study by Kilteni et al. (2019). Our methodological setup differs from that of Kilteni et al. (2019) in some aspects: first, we deviated slightly from the way physical force values were selected in Kilteni et al. (2019). As outlined in “Methods”, our force value selection was based on subjectively perceived estimates rather than physical force intensities. Second, we were unable to directly measure the actual applied intensities and thus could not re-bin trials based on the force measured on the left index finger. In a follow-up study (see supplementary material of Kilteni et al. 2020), it was described that the apparatus from Kilteni et al. (2019) had the opportunity to apply the forces and online tune them based on a feedback controller. The first difference, regarding the subjective determination of forces, should not be considered as critical, since all subjects received the same force values (see our “Methods”). However, we think that the second difference between the setups, the re-binning of trials, might actually explain the stronger results in Kilteni et al. (2019) as this procedure might reduce noise and thereby lead to clearer results. Third, Kilteni et al. (2019) used the same auditory cue for both trial types here. However, the change of 5 exposure and 1 test trials was presented as in the original study. Accordingly, participants also expected a test trial in every sixth trial. Thus, there should be no difference with regard to expectation between our experiments and those of Kilteni et al. (2019). Furthermore, the delay between the first and second touch was randomized between 800 and 1500 ms (Kilteni et al. 2019). We chose a fixed interval of 1500 ms in our experiment, leading to a better prediction of touch. Subjects have been able to reliably predict the occurrence of touch on their finger. Moreover, the authors from the original study (Kilteni et al. 2019) introduced a baseline as a no-movement condition. For the purpose of replication, the 0 ms and the 100 ms test delay were most interesting since in these, the significant differences were found in the original study. In addition, the test subjects’ finger was not fully immobilized during the experiments, which could allow for minor perceptual changes and the applied forces not having the desired magnitude. Lastly, concerning the setup, subjects in the experiment by Kilteni et al. (2019) were asked to fixate a cross at a distance of 2 m during all conditions. The view of their hand and upper arm was impaired by a black screen. In our setup, the left forearm and hand were masked from participants as it was covered by the metal arch. In Experiment 3, we observed effects as reported by Kilteni et al. (2019). After an exposure delay of 0 ms. sensory attenuation was found at a test delay of 0 ms but not at 100 ms, whereas after an exposure delay of 100 ms, the reverse was true, tactile intensity appeared weaker at a 100 ms compared to the 0 ms test delay. A significant interaction effect confirmed the modulation of the test delays by the exposure delays, constant with a temporal recalibration of sensory attenuation. The described differences between our setups might explain why our result are not as strong as those of Kilteni et al. (2019) in Experiment 3. Why was sensory attenuation temporally specific in Experiment 3 and affected test delays rather broadly in the first two experiments? Differences in the experimental setup between Experiment 1, 2 and Experiment 3 concern the presentation of test delays (adaptive staircase vs. constant stimuli), the duration of the touch (270 ms vs. 100 ms) and the number of test delays in the experimental sessions (3 vs. 1). The presentation method of test delays is unlikely to explain the different results. Storch and Zimmermann (2022) showed a successful measurement of sensory attenuation for visual stimuli using the adaptive staircase procedure Best PEST. The duration of the touch being very long in Experiments 1 and 2 might be more likely to be responsible for a temporally broader effect of sensory attenuation. However, a recent study by Kilteni et al. (2023) used a touch duration of 250 ms and did find a difference between 0 and 100 ms test delays. It is hard to believe that the 20 ms difference to the test duration in our first two experiments should produce the different results. The major difference between Experiment 1, 2 and Experiment 3 is the number of test delays. Presenting only a single test delay as in Experiment 3 might allow temporal recalibration narrowly tuned to the singularly probed delay. In this view, a broader range of test delays might likewise lead to a broader tuning of the sensory attenuation time-course. Even though we blocked test delays in Experiment 2, the presentation of different delays in a single session might still have reduced temporal selectivity of sensory attenuation. One possibility for the broad tuning of sensory attenuation might be that presenting several test delays decreases the ability to distinguish their durations. Regression to the average duration is a well-known effect occurring when different durations have to be judged (e.g., Zimmermann and Cicchini 2020). Our results showed no significant difference in sensory attenuation between the two delay conditions of 0 ms and 100 ms in Experiment 1 and 2. This finding led us to exclude the possibility of temporal recalibration effects in these experiments. The lack of differentiation in attenuation between the two delay conditions implies that participants’ perception of the timing between their actions and the resulting sensory feedback remained relatively constant. In Experiment 3, we fixated subject's finger with tape and broadened the range of the subjectively perceived stimulus strength. We observed the well-known phenomenon of sensory attenuation between a test delay of 0 ms and 100 ms in this experiment. The necessity to fixate the finger of subjects suggests that sensory attenuation is highly susceptible for differences between the predicted and the actual force. It is one of the key statements of the first experiments that even small methodological changes might influence the observed effects of sensory attenuation.

The favorite theoretical approach to explain the phenomenon of sensory attenuation involves an internal forward model that predicts the sensory consequences of a button press (Blakemore et al. 2000, 2002). Based on a copy of the motor plan to press the button, the expected intensity of the ensuing tactile sensation and the time of its occurrence will be predicted. If the predicted and the actual sensation match, sensory attenuation will be observed. The content of the predictions is likely shaped by experience, given that signal transductions speeds and motor execution might change across the lifetime. Systematic mismatches between predicted and actual sensations lead to adaptation in many cases, likely to be processed within a forward model (Shadmehr et al. 2010). Recent literature findings also challenged the interpretation of the forward model (Press et al. 2020; Yon et al. 2018, 2021). In these studies, sensory consequences were amplified instead of weakened by the prediction of sensorimotor processes. Representations of visual brain areas changed towards expected action outcomes which makes the explanation of domain-general ideas more plausible (Yon et al. 2018). The authors hypothesize that an increased importance of prediction errors or sensory gating may be responsible for these attenuating effects. It is important to note that expectations can bias our actions towards perceiving expected outcomes, as highlighted by Yon et al. (2021). However, recent literature findings found evidence against this enhancement view for attenuation of self-touch (Job and Kilteni 2023; Kilteni and Ehrsson 2022).

Roussel et al. (2013) suggested a model based on the ideomotor theory, stating that the preparation of a motor movement consists in the preactivation of the sensory consequences of that movement. Brown et al. (2013) offer an explanation of sensory attenuation that reflects the active inference perspective of predictive coding. The theory holds that around the time of movements sensory processing prioritizes the proprioceptive consequences of these movements, thus leading to sensory attenuation in other sensory channels. This account is in agreement with findings of sensory attenuation for visual (Cardoso-Leite et al. 2010; Desantis et al. 2014; Hughes and Waszak 2011; Yon and Press 2017; but see Schwarz et al. 2018) and auditory stimuli (Baess et al. 2009; Weiss et al. 2011). In both models (Brown et al. 2013; Roussel et al. 2013), the time when sensory attenuation starts should be coupled to the onset of the movement since it is claimed that movement preparation factors are responsible for the effect. We have recently demonstrated that the tactile sensation felt in the active hand when pressing a button or a force sensory is necessary for sensory attenuation at the passive finger to occur (Fritz et al. 2022). We argued that attention shifting to the active finger to process the tactile sensation leads to reduced attentional resources in the passive finger, resulting in sensory attenuation. This idea, when applied to sensory attenuation of self-touch, cannot account for a temporal dissociation of the active touch by the subject and the time when sensory attenuation starts. Since the tactile sensation felt during the active touch is never shifted in time, sensory attenuation should also stick to the time of the movement. However, our explanation was proposed for sensory attenuation of external events like visual or auditory events. There are many arguments suggesting that sensory attenuation for external events and for self-touch rely on separate mechanisms (Dogge et al. 2019).

In conclusion, we show in three experiments that the temporal spread of sensory attenuation depends on the temporal range of test delays. We replicated the original report of temporal recalibration of sensory attenuation (Kilteni et al. 2019) in which a single test delay was used. With this experimental design, sensory attenuation was tightly tuned to the exposed delay. In experiments with three test delays presented in one session, we found a much broader tuning of sensory attenuation. Perceptual indistinguishability of the different test delays through regression to the mean might explain the broad temporal spread of sensory attenuation.

## Data Availability

The datasets generated and analyzed during the current study are available in the repository: https://osf.io/thfrj/.
